# The Epidemiology of Dementia in LGBTQ Older Adults

**DOI:** 10.1093/geroni/igaa057.2695

**Published:** 2020-12-16

**Authors:** Jason Flatt

**Affiliations:** University of Nevada, Las Vegas, School of Public Health, Las Vegas, California, United States

## Abstract

Over 3 million or more adults aged 60 + live in the US who identify as lesbian, gay, bisexual, transgender, and/or queer (LGBTQ). Less is known about dementia risk in LGBTQ older adults. We will discuss dementia risk and related risk factors among LGBTQ adults from multiple population-based and cohort studies. We found higher rates of subjective memory problems among lesbian, bisexual and transgender adults compared to both gay men and heterosexual men and women. Using medical record data, 8% (343) of LGB adults aged 60+ were diagnosed with dementia. They were more likely to identify as male (63% vs. 44%), had a higher education level (college degree+ 63% vs. 40%) and were younger than their non-LGB counterparts. These findings highlight dementia risk and related problems among LGBTQ older adults. Future studies are needed to better understand dementia risk and recruiting, screening and improving dementia-related outcomes in LGBTQ older adults.

